# The Use of Bacteria and Their Toxins as Antitumor Agents: Present and Future

**DOI:** 10.3390/microorganisms14050964

**Published:** 2026-04-24

**Authors:** Luz María Ibarra-Velázquez, Marco Antonio Cardona-López, Reynaldo Salvador Cervantes-Figueroa, Alba Guadalupe Ascencio-Navarrate, María Elena Becerra-Mercado, Ana Luisa Madriz-Elisondo

**Affiliations:** Departamento de Ciencias Médicas y de la Vida, Centro Universitario de la Ciénega, Universidad de Guadalajara, Av. Universidad 1115, Lindavista, Ocotlán 47820, Jalisco, Mexico; luz.ibarra@academicos.udg.mx (L.M.I.-V.); marco.cardona@academicos.udg.mx (M.A.C.-L.); reynaldo.cervantes@academicos.udg.mx (R.S.C.-F.); alba.ascencio6643@alumnos.udg.mx (A.G.A.-N.); maria.bmercado@academicos.udg.mx (M.E.B.-M.)

**Keywords:** cancer, oncolytic bacteria, bacterial toxins, immunotoxins, innovative therapies

## Abstract

Cancer remains one of the leading causes of morbidity and mortality worldwide, and despite major advances in surgery, chemotherapy, radiotherapy, and immunotherapy, important therapeutic limitations persist, including systemic toxicity, therapeutic resistance, and poor drug penetration into hypoxic tumor regions. These challenges have renewed interest in alternative biological strategies, particularly the use of bacteria and bacterial toxins as antitumor agents. Certain bacterial species possess intrinsic tumor-targeting properties, including the ability to selectively colonize hypoxic and necrotic regions of solid tumors that are poorly accessible to conventional therapies. This review provides a comprehensive analysis of the mechanisms underlying bacteria-mediated anticancer activity, including selective tumor colonization, direct oncolysis, immune activation, and toxin-mediated cytotoxicity. Both obligate anaerobes (e.g., *Clostridium* and *Bifidobacterium*) and facultative anaerobes (e.g., *Salmonella*, *Escherichia coli*, and *Listeria monocytogenes*) are examined for their tumor-targeting potential. In addition, we discuss the oncological applications of several bacterial toxins and toxin-derived therapeutic constructs, including Cytolysin A (ClyA), *Clostridium difficile* toxin B (TcdB), diphtheria toxin, *Pseudomonas aeruginosa* exotoxin A, and *Clostridium perfringens* enterotoxin (CPE). Emerging strategies such as recombinant immunotoxins and bacterial-directed enzyme prodrug therapy (BDEPT) are also reviewed. Finally, current translational challenges, including pharmacokinetic limitations, immune clearance, and biosafety considerations, are analyzed, highlighting future directions for integrating bacteria-based platforms into next-generation cancer therapies. This approach reflects the growing interest in microbial strategies for oncology and underscores the potential of bacteria and their toxins as innovative tools in the development of targeted anticancer therapies.

## 1. Introduction

Cancer remains one of the leading global health challenges and continues to be a major cause of morbidity and mortality worldwide. Recent epidemiological analyses estimate that approximately 18.1 million new cancer cases and 9.6 million cancer-related deaths occur annually across multiple world regions, highlighting the growing burden of the disease on healthcare systems and societies [1,2,3,4,5]. Lung cancer remains the most frequently diagnosed malignancy and the leading cause of cancer mortality, while breast, prostate, and colorectal cancers represent a substantial proportion of global cancer incidence. Despite continuous advances in conventional treatments, including surgery, chemotherapy, radiotherapy, and targeted therapies, many solid tumors remain difficult to treat due to therapeutic resistance, systemic toxicity, and limited drug penetration into the tumor microenvironment [1,2,3,4,5].

The tumor microenvironment plays a critical role in therapeutic failure. Solid tumors frequently develop regions characterized by hypoxia, necrosis, abnormal vasculature, and elevated interstitial fluid pressure, all of which hinder the effective delivery of therapeutic agents and contribute to tumor progression and treatment resistance [6,7,8]. These barriers have stimulated the exploration of alternative biological strategies capable of overcoming the physical and physiological constraints of tumor tissues.

Among these emerging approaches, the use of bacteria as therapeutic agents has attracted renewed attention. Certain bacterial species possess intrinsic properties that make them particularly suitable for targeting tumors, including preferential colonization of hypoxic and necrotic tumor regions that are poorly accessible to conventional therapies. Both obligate anaerobes, such as *Clostridium* and *Bifidobacterium*, and facultative anaerobes, including *Salmonella*, *Escherichia coli*, and *Listeria monocytogenes*, have demonstrated the ability to selectively accumulate within tumors and induce direct or immune-mediated antitumor effects [9,10,11,12].

The concept of bacterial cancer therapy has historical roots dating back to the pioneering work of William B. Coley in the late nineteenth century, who observed tumor regression following the administration of bacterial toxins in patients with sarcoma [13]. More recently, bacterial-based immunotherapies such as Bacillus Calmette–Guérin (BCG) have become well-established clinical treatments, particularly for non-muscle invasive bladder cancer, where they stimulate local immune responses and contribute to tumor control [14,15]. These historical and clinical observations have provided a strong foundation for the development of modern bacterial-based anticancer strategies.

Advances in synthetic biology and genetic engineering have significantly expanded the therapeutic potential of bacteria. Engineered bacterial platforms can now be designed to enhance tumor specificity, improve safety profiles, and deliver therapeutic payloads such as cytotoxic proteins, immune modulators, or prodrug-converting enzymes directly within the tumor microenvironment [9,10,11,12]. In parallel, bacterial toxins have emerged as valuable components of targeted anticancer therapies, particularly through the development of recombinant immunotoxins and toxin-derived cytotoxic agents capable of selectively eliminating malignant cells.

In this context, bacteria and their toxins represent a promising class of biological agents with unique mechanisms of tumor targeting and destruction. The ability of bacteria to colonize tumor tissues, modulate the immune microenvironment, and deliver potent cytotoxic molecules provides opportunities to complement or enhance existing anticancer therapies.

Therefore, the aim of this review is to provide a comprehensive analysis of the current advances in bacterial-based anticancer strategies, including tumor-targeting bacteria, engineered bacterial platforms, bacterial toxins, immunotoxins, and enzyme-activated prodrug therapies. Additionally, we discuss the mechanistic basis of these approaches, their preclinical and clinical development, and the major challenges that must be addressed to facilitate their translation into effective cancer treatments.

## 2. Reprogramming the Tumor Ecosystem: Clinical Validation of BCG and the Emergence of Precision Bacterial Oncology

Cancer remains one of the leading causes of morbidity and mortality worldwide, with more than 20 million new cases reported in 2022 and projections exceeding 35 million annually by 2050 [1]. Despite substantial advances in surgery, chemotherapy, radiotherapy, targeted therapy, and immunotherapy, advanced solid tumors continue to present major therapeutic challenges, including systemic toxicity, drug resistance, poor intratumoral penetration, and diminished efficacy under hypoxic conditions [4].

Contemporary oncology no longer defines cancer solely as uncontrolled cellular proliferation but rather as a dynamic and evolving ecosystem composed of malignant cells, stromal fibroblasts, immune infiltrates, aberrant vasculature, and remodeled extracellular matrix [5,16]. This tumor microenvironment (TME) generates pathological constraints that directly compromise treatment efficacy. Among the most relevant are hypoxia caused by chaotic vascularization, extensive necrotic regions, chronic inflammation, immune suppression, and elevated interstitial fluid pressure (IFP) [7,8]. Hypoxia is particularly critical, as the cytotoxic effects of radiotherapy depend on oxygen-mediated stabilization of DNA damage, rendering hypoxic regions markedly radioresistant [7,17]. Likewise, abnormal vascular architecture and increased IFP hinder homogeneous drug distribution, creating poorly perfused sanctuary niches that favor tumor survival and therapeutic resistance [8]. Intratumoral heterogeneity and persistent inflammatory signaling further contribute to immune evasion and treatment failure [6].

Paradoxically, these hostile tumor conditions create a biologically permissive niche for certain microorganisms. Obligate and facultative anaerobic bacteria preferentially colonize hypoxic, necrotic, and immunosuppressed environments—precisely the regions where conventional therapies exhibit reduced effectiveness [9,10]. This ecological compatibility has renewed scientific interest in exploiting bacteria and their toxins as innovative anticancer agents. Unlike synthetic drugs, bacteria are living systems capable of active motility, selective tumor colonization, and in situ production of therapeutic molecules. Advances in synthetic biology have enabled virulence attenuation, tumor-specific gene expression, quorum-sensing regulation, and controlled delivery of cytotoxic payloads, transforming bacteria into programmable therapeutic platforms [11,12].

Within this conceptual framework, BCG represents the first clinically validated bacterial immunotherapy for a solid tumor. Although early bacterial cancer therapy is historically associated with Coley’s toxins [13], BCG constitutes the first FDA-approved and clinically established bacterial-based treatment and remains the gold-standard intravesical therapy for non-muscle invasive bladder cancer (NMIBC), with maintenance regimens recommended according to risk stratification [14,15].

BCG therapy follows a coordinated immunobiological sequence. After intravesical instillation, BCG attaches to urothelial and tumor cells through fibronectin bridging α5β1 integrins via its fibronectin attachment protein (FAP), facilitating bacterial internalization. The negatively charged glycosaminoglycan layer of normal urothelium limits excessive adhesion, contributing to localized activity [14]. Internalization activates the Toll-like receptor (TLR2 and TLR4) signaling pathways, leading to the primary response to myeloid differentiation 88 (MyD88)-dependent nuclear factor-kappa B (NF-κB) activation and the transcription of pro-inflammatory cytokines.

This signaling cascade induces a T helper 1 (Th1)-polarized immune milieu characterized by the production of interleukin (IL)-2, IL-6, IL-8, interferon (IFN)-γ, and tumor necrosis factor (TNF)-α. Neutrophils dominate the early inflammatory infiltrate and mediate tumor cell killing through release of tumor necrosis factor related apoptosis inducing ligand (TRAIL) and neutrophil extracellular trap formation. Macrophages, natural killer (NK) cells, and cluster of differentiation 8 (CD8^+^) T lymphocytes contribute to sustained adaptive antitumor immunity [14,15]. In parallel, direct tumor cytotoxicity—including apoptotic and caspase-independent necrotic pathways associated with oxidative stress and High-Mobility Group Box 1 (HMGB1) release—may reduce tumor burden before full adaptive activation is established.

Despite its established clinical efficacy, 30–40% of patients exhibit refractory disease. Mechanistic analyses suggest that BCG-induced Mitogen-Activated Protein Kinase (MAPK)/NF-κB signaling can promote programmed death ligand 1 (PD-L1) upregulation on tumor cells, contributing to adaptive immune resistance and providing a rationale for combination strategies with immune checkpoint inhibitors [14,15]. Variability among BCG substrains and treatment-related toxicity further underscore the need for improved bacterial engineering strategies.

The clinical validation of BCG establishes a foundational paradigm for localized bacterial immunotherapy. Building upon this precedent, contemporary engineered bacterial platforms aim to enhance tumor-selective colonization, optimize genetic attenuation, incorporate programmable regulatory circuits, and deliver therapeutic payloads with improved safety and specificity. In this context, the very biological constraints that limit conventional oncologic therapies hypoxia, necrosis, immune suppression, and impaired drug penetration may represent exploitable vulnerabilities when approached through rationally engineered bacterial-based strategies.

**Figure 1:**
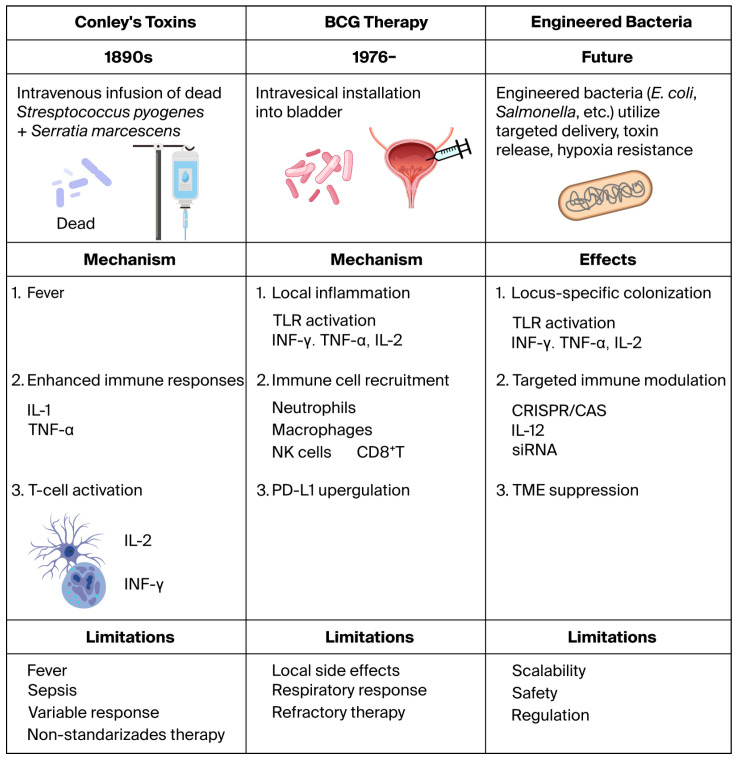
Evolution of bacterial-based cancer therapies, from Coley’s toxins (1890s), which induced fever and systemic immune activation, to intravesical BCG therapy (first clinical report in 1976; FDA approval in 1990), characterized by localized inflammation, immune recruitment, and PD-L1 upregulation, and finally to engineered bacteria designed for tumor-specific colonization and targeted immune modulation (e.g., CRISPR/Cas systems, cytokine delivery platforms). Key limitations, including systemic toxicity, local adverse effects, safety concerns, and regulatory challenges, are indicated for each stage.

Bacterial-based cancer therapy has progressed from empirical systemic immune stimulation to targeted and genetically engineered approaches. Coley’s toxins induced fever and broad immune activation through intravenous administration of inactivated bacteria. BCG therapy introduced localized intravesical immunomodulation characterized by TLR activation, immune cell recruitment, and programmed death ligand 1 (PD-L1) upregulation. Current engineered bacteria are designed for tumor-specific colonization and targeted immune modulation, although safety and scalability remain important challenges. Figure 1 summarizes the evolution of bacterial-based cancer therapies.

## 3. Mechanisms of Tumor-Targeting and Tumoricidal Activity of Bacteria

Cancer treatment based on bacteria relies on their capacity to selectively accumulate within tumors and exert multiple tumoricidal mechanisms at cellular, molecular, and immunological levels. Unlike conventional therapies, bacteria exploit the distinctive characteristics of the TME, including hypoxia, necrosis, aberrant vasculature, and local immune suppression, which facilitate preferential colonization of malignant tissues [9,10,18].

One of the principal mechanisms underlying tumor selectivity is hypoxia-dependent colonization. Obligate anaerobes such as *Clostridium* and *Bifidobacterium* germinate exclusively in anoxic regions of solid tumors (≤10 mmHg oxygen), where rapid tumor proliferation exceeds vascular oxygen supply [19,20]. This strict metabolic requirement prevents their survival in normoxic healthy tissues, thereby conferring intrinsic tumor specificity. In contrast, facultative anaerobes such as *Salmonella typhimurium* and *Escherichia coli* utilize additional mechanisms including chemotaxis toward tumor-derived metabolites, entrapment within disorganized tumor vasculature, and preferential proliferation in inflammatory microenvironments [12,18,20]. Genetic attenuation strategies, such as auxotrophic mutations and deletion of virulence-associated genes, further enhance biosafety by restricting replication in normal organs while preserving tumor tropism [18,21].

Beyond selective colonization, bacteria exert direct tumoricidal effects through cellular lysis and apoptosis induction. Oncolytic strains replicate within tumor tissue, leading to mechanical disruption of malignant cells and release of tumor-associated antigens [21]. This process not only contributes to tumor mass reduction but also stimulates innate and adaptive immune responses, promoting dendritic cell activation, cytotoxic T lymphocyte priming, and long-term antitumor immunity [22,23]. The liberation of damage-associated molecular patterns (DAMPs) amplifies immune recognition and reinforces systemic antitumor effects.

At the molecular level, bacterial toxins represent key mediators of tumor cell destruction. Pore-forming toxins such as ClyA disrupt cellular membranes and induce apoptosis in tumor models [18,24]. Other toxins inhibit essential intracellular processes: diphtheria toxin and *Pseudomonas aeruginosa* exotoxin A block protein synthesis via inactivation of elongation factor-2, leading to apoptotic cell death [25,26], while anthrax lethal factor interferes with mitogen-activated protein kinase (MAPK) signaling pathways through zinc-dependent metalloprotease activity, impairing cell survival signaling [27,28]. Cyclomodulins such as cytolethal distending toxins (CDTs) arrest the cell cycle and inhibit tumor proliferation [29]. Furthermore, TcdB has demonstrated the capacity to inhibit B-cell lymphoma 2 (Bcl-2) expression, induce apoptosis, activate inflammatory antitumor responses, and significantly reduce tumor volume in preclinical breast cancer models [30,31,32].

In addition to direct cytotoxicity, bacteria function as highly specific therapeutic delivery vectors within the tumor microenvironment. Engineered strains can express cytotoxic peptides, therapeutic proteins, cytokines, or enzymes that convert systemically administered prodrugs into active cytotoxic compounds exclusively within tumor tissue [20,33]. This strategy enhances local drug concentration while minimizing systemic toxicity. For example, attenuated *Listeria monocytogenes* strains engineered to express tumor-associated antigens have been evaluated as therapeutic vaccines, illustrating the dual capacity of bacteria to act both as direct tumoricidal agents and as targeted gene delivery platforms [18,34].

Another key consideration is bacterial motility, which allows bacteria to penetrate deeply into tumor tissues. Unlike chemotherapeutic drugs, which rely on passive distribution and often have limited penetration into the inner regions of tumors, bacteria are living organisms capable of generating their own energy from the surrounding microenvironment. This grants them an almost unlimited capacity to migrate and reach even the most inaccessible tumor regions. After systemic administration, bacteria use self-propulsion to move actively away from blood vessels and disperse throughout the tumor tissue. This motility not only enables them to overcome physical barriers that hinder conventional treatments but also increases the likelihood of reaching tumor regions that are typically difficult for drugs to access. As such, bacterial motility represents a significant advantage in the search for more effective cancer therapies [9].

Finally, bacterial therapy may synergize with conventional treatments such as radiotherapy. Tumor-resident bacteria can enhance intratumoral necrosis, promote thrombosis within aberrant tumor vasculature, and help overcome hypoxia-associated radioresistance [7,35]. Their intrinsic motility enables deep penetration into tumor cores that are poorly accessible to chemotherapeutic agents, addressing one of the major limitations of standard anticancer therapies [7,9].

Collectively, these mechanisms promote selective hypoxia-driven colonization, direct oncolysis, immune activation, molecular toxin-mediated cytotoxicity, vector-based therapeutic delivery, and synergy with radiotherapy position bacteria as multifaceted antitumor agents capable of exerting both direct and indirect tumoricidal effects within complex tumor ecosystems [10,11,18].

**Figure 2:**
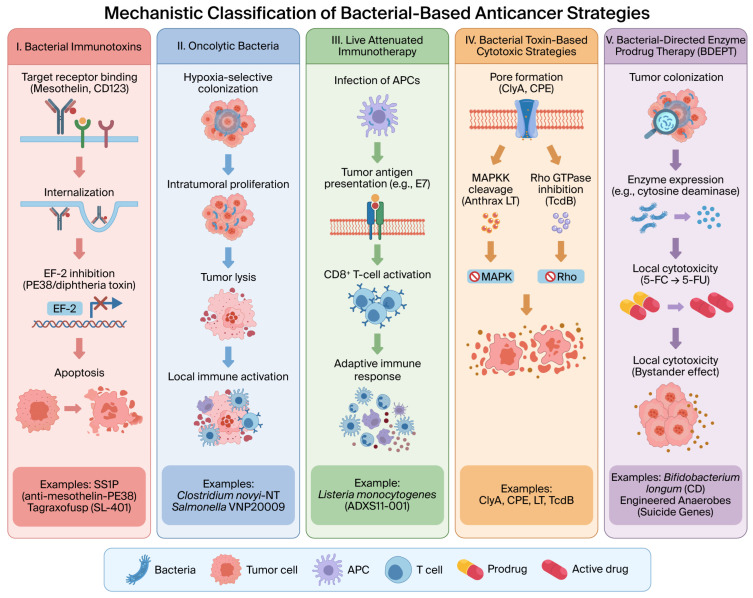
Mechanistic classification of bacterial-based anticancer strategies. (I) Bacterial immunotoxins induce receptor-targeted cytotoxicity via internalization and EF-2 inhibition. (II) Oncolytic bacteria selectively colonize tumors, promote lysis, and activate local immunity. (III) Live attenuated platforms enhance antigen presentation and CD8^+^ T-cell-mediated adaptive responses. (IV) Bacterial toxin-based strategies directly kill tumor cells through pore formation or inhibition of key signaling pathways. (V) Bacterial-directed enzyme prodrug therapy (BDEPT) enables intratumoral prodrug conversion and localized cytotoxicity via a bystander effect.

Figure 2 and Table 1 summarize representative bacterial-derived anticancer strategies classified according to their principal mechanistic categories and stage of development. These include recombinant bacterial immunotoxins, oncolytic bacteria, live attenuated immunotherapeutic platforms, toxin-based cytotoxic approaches, and bacterial-directed enzyme prodrug therapy (BDEPT). Together, these examples illustrate the mechanistic diversity and translational potential of bacteria and bacterial toxins in cancer therapy.

## 4. Possible Synergistic Role of Bacteria/Toxins with Existing Combination Therapies

- Bacteria/Toxin-Based Therapies in Combination with Immunotherapy

An important emerging direction in bacterial-based oncology is their potential synergistic integration with immunotherapy. Mechanistically, oncolytic and engineered bacteria can enhance antitumor immune responses by inducing localized necrosis, intratumoral thrombosis, and immunogenic cell death, thereby increasing tumor antigen release and promoting immune priming within the tumor microenvironment [7]. These effects may contribute to the conversion of immunologically “cold” tumors into more inflamed and T-cell-infiltrated lesions, potentially improving responsiveness to immune checkpoint blockade. Furthermore, growing evidence indicates that both the gut and intratumoral microbiome significantly influence the efficacy of immune checkpoint inhibitors, positioning bacterial modulation as a predictive biomarker and therapeutic adjuvant in immunotherapy [23]. Preclinical studies also suggest that engineered bacteria can reshape the tumor immune microenvironment by reducing immunosuppressive myeloid-derived suppressor cells and enhancing CD8^+^ T-cell activation, thereby reinforcing antitumor immunity and potentially potentiating PD-1/PD-L1 blockade [12]. However, despite these promising synergistic mechanisms, combination strategies must consider possible limitations, including excessive inflammatory activation, immune-related adverse events, and accelerated immune-mediated bacterial clearance, which may compromise sustained intratumoral colonization. Thus, while bacterial immunotherapy combinations offer substantial translational promise, careful optimization of dosing, strain attenuation, and immune monitoring will be essential to ensure therapeutic balance.

- Bacteria/Toxin-Based Therapies in Combination with Radiotherapy

Radiotherapy (RT) represents a rational and potentially synergistic partner for bacterial- and toxin-based antitumor strategies. One of the expected synergistic effects arises from the preferential colonization of hypoxic tumor regions by tumor-targeting bacteria, which coincide with areas of intrinsic radioresistance. Engineered bacterial platforms capable of modulating tumor oxygenation such as catalase-expressing strains have demonstrated the ability to alleviate hypoxia, enhance reactive oxygen species (ROS) generation, and increase RT-induced DNA damage, thereby improving radiosensitivity in preclinical models [53]. In addition, direct experimental evidence shows that combining engineered *Salmonella typhimurium* with RT results in enhanced tumor suppression compared to either modality alone, accompanied by increased DNA damage and modulation of intratumoral immune infiltration [54]. These findings support the concept that bacterial tumor tropism and metabolic engineering can complement the cytotoxic and immunogenic effects of radiotherapy.

However, potential drawbacks must also be considered. Radiotherapy can alter host microbiota composition and disrupt epithelial barrier integrity, particularly in abdominal or pelvic irradiation, potentially increasing inflammatory toxicity and contributing to variability in therapeutic response [55]. Moreover, the combination of radiation-induced tissue damage with bacterial colonization may amplify local inflammation, raising concerns regarding safety and tolerability. Therefore, while RT–bacteria/toxin combinations hold promise for radiosensitization and immune potentiation, careful optimization of bacterial strain attenuation, dose scheduling, and toxicity monitoring will be critical for safe clinical translation.

- Bacteria/Toxin-Based Therapies in Combination with Chemotherapy

Combining bacteria- or bacteria-derived platforms with chemotherapy offers a mechanistically strong route to synergy by enhancing intratumoral drug delivery while potentially reducing systemic exposure. Tumor-targeting bacteria can actively colonize hypoxic and poorly perfused regions that are often difficult to reach with conventional chemotherapy, thereby improving drug penetration and spatial distribution within solid tumors [56]. In addition, engineered bacterial systems can function as programmable carriers for chemotherapeutics, enabling co-delivery of bacterial effectors and cytotoxic drugs to achieve additive or synergistic tumor control. For example, an engineered bacterial delivery system carrying doxorubicin-loaded nanoparticles demonstrated enhanced melanoma suppression and reduced side effects compared with less targeted approaches, supporting the concept of bacteria-mediated spatiotemporally controlled chemotherapy delivery. However, important drawbacks must be considered in a review context. Chemotherapy-related immunosuppression may increase infection risk and complicate safety when using live biotherapeutics, while systemic clearance, biodistribution variability, and complex host–bacteria interactions remain translational hurdles [57]. Therefore, although bacteria/derivative–chemotherapy combinations are promising for improving tumor targeting and therapeutic index, careful attenuation strategies, dosing schedules (avoiding peak neutropenia), and safety controls will be essential for clinical translation [58].

## 5. Safety Challenges of Live Bacterial Cancer Therapy and Quorum-Based Biocontention

**Figure 3:**
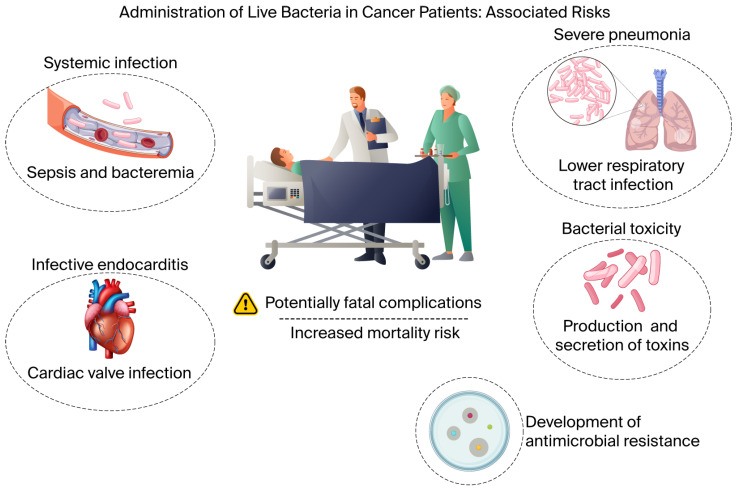
Risks associated with live bacterial therapy in cancer patients. Live bacterial administration may cause systemic infection (bacteremia and sepsis), infective endocarditis, severe pneumonia, toxin-mediated toxicity, and the emergence of antimicrobial resistance, potentially resulting in life-threatening complications and increased mortality.

The clinical application of live bacteria in cancer therapy entails inherent safety risks, particularly in patients with advanced malignancies who are frequently immunocompromised as a consequence of the disease itself or prior anticancer treatments. Evidence from early-phase clinical trials has demonstrated that, despite genetic attenuation, the administration of live bacteria may still result in clinically significant adverse events. In a Phase I study evaluating the intravenous administration of attenuated *Salmonella typhimurium* (VNP20009) in patients with metastatic melanoma, dose-limiting toxicities and systemic inflammatory responses were reported, underscoring the narrow therapeutic window associated with systemic delivery of live bacterial agents [39]. Similarly, a Phase I trial involving intratumoral injection of *Clostridium novyi*-NT spores in patients with treatment-refractory solid tumors documented severe local and systemic toxicities related to bacterial germination and tumor lysis, highlighting that even localized delivery strategies do not fully eliminate safety concerns. Collectively, these clinical observations emphasize that the introduction of live bacteria into cancer patients carries non-negligible risks, particularly in immunocompromised populations, and reinforce the necessity of stringent patient selection, intensive safety monitoring, and the continued refinement of attenuation and biocontainment strategies to mitigate the likelihood of catastrophic infectious outcomes. Figure 3 summarizes the principal clinical risks associated with the administration of live bacteria in cancer patients.

Beyond permanent genetic attenuation, quorum-based regulatory strategies have emerged as particularly suitable approaches for controlling the use of bacteria and their toxins as antitumor agents, both in current applications and future developments. Quorum sensing-controlled systems enable the population-dependent activation of therapeutic functions, allowing bacteria to express cytotoxic or immunomodulatory toxins preferentially within the tumor microenvironment, where bacterial density and local cues converge. This strategy permits the effective exploitation of bacterial toxins for antitumor activity while minimizing their expression in systemic tissues, thereby reducing off-target toxicity and systemic virulence [16,59]. From a safety-oriented perspective, quorum quenching (quorum extinction) represents a complementary antivirulence layer by disrupting quorum-dependent signaling pathways responsible for the coordinated activation of pathogenic traits, without necessarily compromising bacterial viability [16]. The integration of quorum sensing-regulated toxin delivery with quorum quenching-based suppression of systemic virulence provides a rational framework for the development of programmable bacterial therapeutics capable of selectively attacking tumors while avoiding catastrophic infectious risks, particularly in immunocompromised cancer patients. Collectively, these quorum-based control strategies align with the present use and future potential of bacteria and their toxins as controllable and precise antitumor agents.

## 6. Therapeutic Potential of Bacterial Toxins in Oncology

The intrinsic lethality of bacterial toxins, a product of refined coevolution with host organisms, represents one of the most promising attributes in biopharmaceutical development. Current bioengineering strategies focus on designing delivery systems that maximize cytotoxic selectivity, allowing these proteins to act as highly precise antitumor agents while preserving the integrity of healthy tissues [20].

As primary virulence factors, protein toxins have evolved to subvert fundamental cellular processes, facilitating invasion and evasion of the immune system [18]. This capacity for cell manipulation is the basis of their oncological application:

- Cell Cycle Modulation: Cytolethal stretching toxins (CDTs) and cell cycle inhibitory factor (CIF) act as potent inhibitors of mitosis. Although these mechanisms can interfere with the clonal expansion of lymphocytes, their ability to arrest the cell cycle offers a pathway for controlling malignant proliferation. In contrast, factors such as cytotoxic necrotizing factor (CNF) allow for exploring the manipulation of cell differentiation and cycle progression in specific contexts [29].
- Induction of Apoptosis and Lysis: Cytolysin A (ClyA or HlyE) stands out as a pore-forming toxin capable of disrupting the cytoplasmic membrane and inducing apoptosis. The use of bacterial vectors such as *Escherichia coli* or *Salmonella typhimurium* engineered to express ClyA has demonstrated a significant reduction in tumor burden in in vivo models, validating its potential in antitumor gene therapy [18,24].

Even highly pathogenic systems, such as those produced by *Bacillus anthracis*, exhibit antitumor properties after molecular characterization. Lethal toxin (LeTx), resulting from the interaction between protective antigen (PA) and lethal factor (LF), acts as a zinc metalloproteinase that inactivates isoforms of the MAPK pathway. This disruption of survival signaling pathways has exhibited remarkable efficacy against melanoma in preclinical settings, while edema toxin (EdTx) alters cellular water balance by causing a supraphysiological elevation of cAMP [28].

Breast cancer remains one of the most prevalent and deadly cancers in the Western world, frequently facing the challenge of chemoresistance [2,60]. In this context, *Clostridium difficile* toxin B (CdtB) emerges as a bimodal therapeutic tool:

- Direct Cytotoxic Activity: CdtB acts as a cytotoxic virulence factor that inhibits cell proliferation [31,61] and triggers programmed cell death or necrosis in various breast cancer cell lines [32].
- Tumor Microenvironment Immunomodulation: In addition to its proteolytic effect, CdtB induces the secretion of proinflammatory chemokines and cytokines [30,62]. Experimental findings confirm that this toxin not only reduces tumor volume through direct cytotoxicity but also stimulates a robust immune response against the neoplasm, solidifying its profile as a cutting-edge therapeutic strategy [18,23].

Shiga toxins (Stx1 and Stx2) are AB5 ribosome-inactivating exotoxins produced by *Shigella dysenteriae* and enterohemorrhagic *Escherichia coli*. Their antitumor potential is primarily determined by the expression of the globotriaosylceramide receptor (Gb3/CD77), which mediates toxin binding and internalization. Upon receptor engagement, Stx undergoes endocytosis and retrograde trafficking to the endoplasmic reticulum, where the enzymatically active A subunit translocates into the cytosol and depurinates 28S rRNA, leading to irreversible inhibition of protein synthesis and activation of ribotoxic stress pathways [63,64,65]. Tumor-associated overexpression of Gb3 has been documented in several malignancies. Notably, pancreatic and colorectal adenocarcinomas exhibit significantly increased Gb3/CD77 expression compared with non-neoplastic tissues, rendering these tumors susceptible to Stx-mediated cytotoxicity [66]. More recently, in vitro studies in triple-negative breast cancer demonstrated Gb3-dependent internalization of Stx2, resulting in reduced cell viability, impaired proliferation and migration, and induction of autophagy [67]. These findings indicate that Gb3 overexpression represents a critical determinant for tumor selective targeting by Stx. However, despite receptor-driven selectivity at the cellular level, the intrinsic systemic toxicity of the holotoxin responsible for severe endothelial damage and hemolytic uremic syndrome remains a major obstacle for clinical translation, supporting ongoing investigation into engineered variants and subunit-based delivery platforms [63].

Shiga toxin-based therapies remain in early clinical phases (I/II). Progress toward FDA approval currently depends on the validation of deimmunization strategies and the success of neutralizing agents such as INM004 in mitigating systemic risks [68]. Although these toxins originally evolved to weaken the host’s immune response, their ability to deregulate essential cell survival pathways makes them cutting-edge candidates for the development of targeted drugs aimed at eradicating tumors resistant to conventional treatments.

## 7. Immunotoxins: Chimeric Protein Engineering in Cancer Immunotherapy

Immunotoxins represent one of the most sophisticated strategies in contemporary precision medicine. These chimeric molecules, designed through bioengineering, combine a targeting domain (typically a monoclonal antibody or a fragment thereof) with an effector protein toxin. This design allows for the specific recognition of surface antigens on neoplastic cells, facilitating the internalization of the toxin via receptor-mediated endocytosis, thus minimizing cytotoxicity in healthy systemic tissues [69].

Once in the cytosol, the cytotoxic component exerts its action, commonly through the irreversible inhibition of protein synthesis, triggering a signaling cascade that culminates in apoptosis. The evolution of these molecules has gone through several critical stages to mitigate biological limitations:

- Optimization of Immunogenicity: The first generations, based on murine antibodies, exhibited high immunogenicity that limited their clinical efficacy. Currently, fourth-generation immunotoxins employ humanized antibodies and modified toxin variants to reduce the antichimeric antibody response, significantly improving the safety profile and plasma half-life [25].
- Synergy with Immunotherapy: Immunotherapy has transformed the oncology paradigm by enhancing the intrinsic capacity of the immune system to identify malignant phenotypes. However, immune evasion and mimicry with self-tissue remain major obstacles. In this context, the use of bacterial vectors and recombinant toxins acts as a molecular adjuvant, increasing the tumor’s immunogenicity and making it more “visible” to host effector cells [70].

In the field of recombinant immunotoxins, the ligand (antibody or growth factor) and the toxin are encoded in a single DNA sequence and expressed in heterologous systems, which are generally bacterial. This molecular architecture allows for high-affinity targeting of specific antigens [69,71].

### 7.1. The Case of Denileukin Diftitox (Ontak^®^)

Hematological malignancies have been the field of greatest clinical success for these molecules. A key example is Denileukin Diftitox (DAB [389]-IL-2), a fusion protein that combines human interleukin-2 (IL-2) with a truncated fragment of diphtheria toxin [20,25]:

- Ligand–Receptor Interaction: The IL-2 portion selectively binds to high-affinity receptors expressed on malignant cells, which function as the target antigen.
- Effector Mechanism: After binding and subsequent internalization, diphtheria toxin inhibits cellular protein synthesis, inducing cell death.
- Clinical Status: This therapy, approved for cutaneous T-cell lymphoma (CTCL), has shown promising activity in leukemias and other lymphomas, and is currently in Phase III clinical trials to solidify its position as a robust therapeutic alternative.

### 7.2. Loncastuximab Terisina

An antibody–drug conjugate (ADC) is a targeted therapeutic platform composed of a monoclonal antibody covalently linked to a highly potent cytotoxic payload through a chemical linker that enables intracellular drug release following antigen binding and internalization [72]. This modular architecture allows selective delivery of cytotoxic agents to tumor cells while minimizing systemic toxicity.

Beyond classical bacterial protein toxins, oncology has evolved toward ADC-based strategies. An illustrative example is Loncastuximab tesirine, an anti-CD19 ADC that received accelerated approval from the U.S. Food and Drug Administration for relapsed or refractory diffuse large B-cell lymphoma. Although it does not contain a bacterial protein toxin, its cytotoxic payload (SG3199) belongs to the pyrrolobenzodiazepine (PBD) class, originally identified as secondary metabolites from Streptomyces species. PBD compounds bind within the minor groove of DNA and induce covalent interstrand cross-links, resulting in potent cytotoxicity [73].

In a Phase II study published in The Lancet Haematology, the combination of loncastuximab tesirine with rituximab demonstrated high overall and complete response rates in patients with relapsed or refractory follicular lymphoma, including high-risk subgroups. The safety profile was manageable and consistent with previous reports, supporting this chemotherapy-free targeted regimen as a clinically relevant therapeutic option in this setting [74].

### 7.3. Moxetumomab Pasudotox

Moxetumomab pasudotox is a recombinant anti-CD22 immunotoxin that incorporates a modified fragment of *Pseudomonas aeruginosa* exotoxin A for targeted cytotoxic delivery in B-cell malignancies. In a pivotal multicenter trial involving patients with relapsed or refractory hairy cell leukemia, moxetumomab pasudotox demonstrated durable clinical activity, with high rates of complete response and achievement of minimal residual disease negativity, supporting its regulatory approval [75]. The mechanism of action involves CD22 binding, internalization, and intracellular inhibition of elongation factor-2, resulting in suppression of protein synthesis and apoptosis. As summarized by Kang (2021) [76], the agent received FDA approval based on its durable response rates and manageable safety profile. Collectively, these data establish moxetumomab pasudotox as a clinically validated example of a bacterial protein toxin successfully engineered into a targeted antitumor therapeutic platform.

### 7.4. Gemtuzumab Ozogamicin

The clinical validation of gemtuzumab ozogamicin (GO) exemplifies the present evolution of bacterial-derived cytotoxic strategies in oncology. GO is an antibody–drug conjugate targeting CD33 that has demonstrated clinically meaningful benefit in acute myeloid leukemia (AML). In the Phase III ALFA-0701 trial, the addition of GO to standard induction chemotherapy significantly improved event-free survival compared with chemotherapy alone in patients with de novo AML, supporting its re-approval under fractionated dosing [77]. More recently, the randomized Phase III AMLSG 09-09 study confirmed the benefit of incorporating GO into intensive chemotherapy in patients with NPM1-mutated AML, demonstrating improved relapse-related outcomes in this molecularly defined subgroup [78]. Furthermore, the UK NCRI AML18 trial provided additional evidence regarding optimal dosing strategy, showing that fractionated administration of GO was associated with improved outcomes and better tolerability in older patients compared with single-dose regimens [79]. Collectively, these randomized Phase III trials consolidate GO as a clinically validated antibody–drug conjugate across different AML risk groups and treatment settings, reinforcing the contemporary relevance of antibody-directed cytotoxic delivery platforms within the framework of bacterial-derived antitumor agents.

## 8. Prodrug Therapies Activated Enzymatically

This strategy leverages genetically modified anaerobic bacteria to selectively combat cancer. These bacteria are engineered to produce an enzyme capable of converting an initially harmless prodrug into a toxic compound. When the bacteria proliferate in tumor regions characterized by necrosis and hypoxia, which are common features of solid tumors, the enzyme is expressed exclusively within this specific environment. Thus, upon systemic administration of the prodrug, activation occurs only inside the tumor, where it is converted into a cytotoxic agent, thereby minimizing damage to healthy tissues and enhancing treatment precision [20].

This type of therapy offers significant advantages compared with conventional chemotherapy. Its main strength lies in its ability to achieve high concentrations of the active compound directly within the target tissue, reducing treatment associated side effects. Moreover, the localized action increases therapeutic efficacy, enabling more aggressive and specific treatments. However, tissue specificity depends on both the therapeutic enzyme used and the vector employed for gene transfer. Ideally, the enzyme should be expressed exclusively in the desired tissue and must efficiently metabolize large quantities of prodrug in the tumor. For this purpose, non-mammalian enzymes are commonly used, although their immunogenic nature presents a challenge. This immune response can be beneficial by inducing antitumor activity or detrimental, depending on the clinical context. Another important consideration is the “bystander effect,” in which neighboring cells that do not express the therapeutic gene can still be affected by the resulting cytotoxic compounds [20,33]. A representative example of this strategy is the use of *Bifidobacterium longum* APS001F engineered to express cytosine deaminase, enabling the intratumoral conversion of the non-toxic prodrug 5-fluorocytosine into the active chemotherapeutic agent 5-fluorouracil within hypoxic tumor regions [20].

## 9. Pharmacokinetic and Mechanical Limitations of the Use of Bacteria and Bacterial-Derived Agents in Cancer Therapies

- Rapid Clearance by the Reticuloendothelial System (RES).

**Figure 4:**
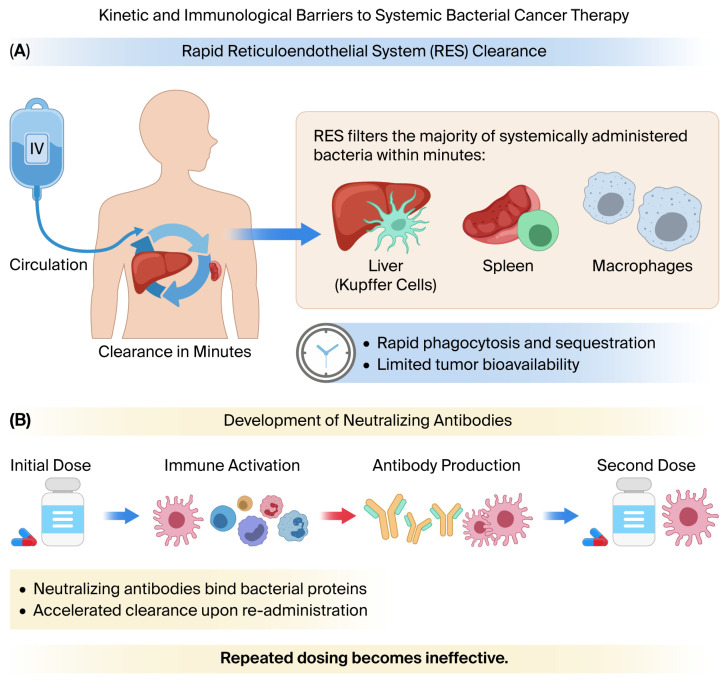
Kinetic and immunological barriers to systemic bacterial cancer therapy. (**A**) Following intravenous administration, therapeutic bacteria are rapidly cleared from circulation by the reticuloendothelial system (RES), including liver Kupffer cells, splenic macrophages, and other phagocytic populations, resulting in limited tumor bioavailability. (**B**) Initial bacterial exposure activates the adaptive immune response, leading to production of neutralizing antibodies that bind bacterial proteins. Upon re-administration, antibody-mediated opsonization accelerates clearance, reducing therapeutic persistence and limiting repeated dosing efficacy.

Following systemic administration, the pharmacokinetic behavior of therapeutic bacteria is strongly influenced by the host mononuclear phagocyte system (MPS). Experimental and clinical studies have demonstrated that a substantial fraction of intravenously administered bacteria is rapidly sequestered in filtration organs such as the liver and spleen, thereby limiting the proportion that reaches tumor tissue. In preclinical tumor models, systemically injected *Salmonella typhimurium* exhibited early hepatic and splenic accumulation with only sparse initial entrapment within tumors, leading to heterogeneous intratumoral distribution [80]. Moreover, clinical evaluation of attenuated *Salmonella* in metastatic melanoma patients confirmed rapid systemic clearance and limited tumor colonization after intravenous administration [39]. Comprehensive reviews further emphasize that innate immune recognition, opsonization, and phagocytic uptake constitute major determinants of bacterial biodistribution and therapeutic efficiency [81]. Collectively, these findings support the notion that rapid RES-mediated clearance represents a critical pharmacokinetic limitation in bacterial anticancer therapy and must be considered when designing systemic delivery strategies (Figure 4).

- Development of Neutralizing Antibodies Against Bacterial Proteins and Toxins.

Bacterial proteins and toxin-derived therapeutic constructs are inherently immunogenic and can induce robust humoral responses following systemic exposure. Clinical and translational studies involving recombinant immunotoxins derived from bacterial exotoxins have demonstrated that patients frequently develop anti-drug antibodies (ADAs) capable of neutralizing the biological activity of the therapeutic agent, thereby significantly reducing efficacy upon repeated dosing [82]. Similarly, clinical experience with immunotoxin-based anticancer therapies has shown that neutralizing antibodies can limit treatment cycles and necessitate modifications in dosing strategies or molecular redesign. The immunogenic nature of bacterial toxin components has been extensively characterized, highlighting the need for epitope modification, humanization strategies, or conditional activation systems to mitigate immune recognition [69]. Therefore, the potential for rapid antibody-mediated neutralization represents a biologically plausible and clinically documented limitation of repeated administration of bacterial toxin-based therapeutics.

- Dense Fibrotic Stroma and Elevated Interstitial Fluid Pressure (IFP).

**Figure 5:**
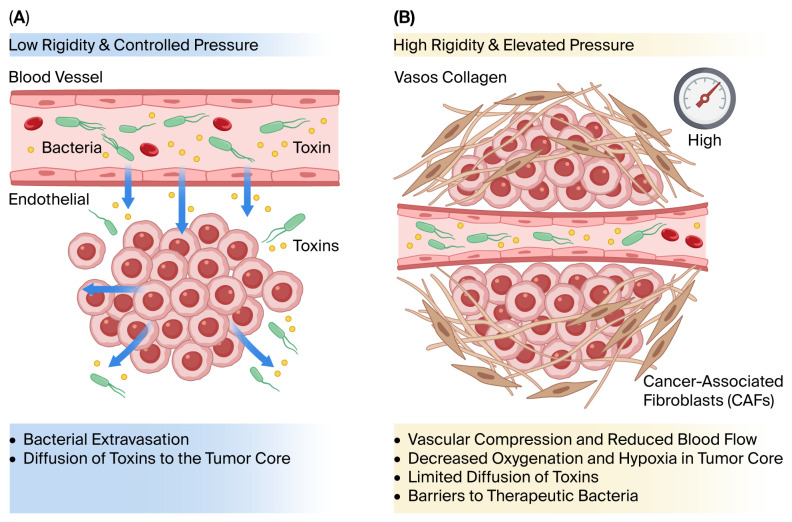
Stromal rigidity and interstitial fluid pressure as intratumoral barriers to bacterial therapy. (**A**) Tumors with low stromal rigidity and controlled interstitial fluid pressure permit efficient bacterial extravasation and toxin diffusion toward the tumor core. (**B**) Highly fibrotic tumors characterized by excessive collagen deposition and cancer-associated fibroblast (CAF) activation exhibit vascular compression and elevated IFP, resulting in reduced perfusion, hypoxia, and limited intratumoral penetration of therapeutic bacteria and toxins.

Beyond systemic pharmacokinetic constraints, the tumor microenvironment itself presents significant biophysical barriers to the effective penetration of therapeutic bacteria and bacterial-derived agents. Many solid tumors exhibit dense desmoplastic stroma characterized by excessive extracellular matrix deposition and abnormal vascular architecture, which contribute to elevated interstitial fluid pressure (IFP). High IFP impairs transvascular transport and reduces convective movement of therapeutic agents from the bloodstream into the tumor interstitium [8]. Additionally, abnormal and compressed tumor vasculature further compromises perfusion and distribution, limiting homogeneous intratumoral delivery [83]. Experimental evidence in pancreatic ductal adenocarcinoma has demonstrated that enzymatic modulation of stromal components can significantly enhance therapeutic penetration, underscoring the restrictive role of fibrotic barriers [84]. These data indicate that successful bacterial cancer therapy must overcome not only immune clearance but also the mechanical and fluid dynamic constraints imposed by the tumor microenvironment (Figure 5).

## 10. Overview of Current Clinical Trials

The clinical translation of bacteria-based cancer therapies and bacterial toxin-derived strategies remains at an early but steadily advancing stage. Current clinical trials primarily focus on malignancies characterized by poor prognosis, hypoxic tumor microenvironments, or resistance to conventional therapies. Among solid tumors, colorectal cancer, pancreatic cancer, melanoma, glioblastoma, advanced breast cancer, and sarcomas are the most frequently investigated indications, owing to their accessibility to bacterial colonization and high unmet clinical need [7,10,20].

Several clinical studies have explored the use of genetically attenuated bacteria as therapeutic agents or delivery platforms. Modified *Salmonella typhimurium* strains have been evaluated in early-phase clinical trials for metastatic melanoma and colorectal cancer, demonstrating preferential tumor targeting with manageable safety profiles, although therapeutic efficacy remains limited when tested as monotherapy [9,10]. Similarly, *Clostridium novyi*-NT spores have been tested in Phase I clinical trials involving patients with advanced solid tumors, including carcinomas and sarcomas. Intratumoral administration resulted in tumor necrosis and local immune activation, while also underscoring the importance of strict control of inflammatory toxicity and bacterial dissemination [19,20].

In parallel, toxin-based strategies have reached more advanced stages of clinical evaluation. Immunotoxins such as denilleukin diftitox (Ontak), approved for the treatment of cutaneous T-cell lymphoma, represent a successful example of the clinical translation of bacterial toxins when combined with precise targeting mechanisms [26]. Additional recombinant immunotoxins targeting hematological malignancies, including leukemias and lymphomas, have progressed through Phase I and II trials, with several studies approaching late-stage clinical evaluation [69,71]. In contrast, enzymatically activated prodrug therapies and engineered oncolytic bacteria largely remain in early clinical development, with most ongoing studies expected to report primary safety and feasibility outcomes within the next 3–5 years [19,51].

Collectively, the outcomes of these trials are expected to play a decisive role in shaping the future landscape of cancer therapy [85]. Positive safety and efficacy signals may support the integration of bacteria-based approaches as complementary strategies to existing treatments, particularly immunotherapy and radiotherapy, for hypoxic or treatment-resistant tumors [81,86]. Moreover, the clinical validation of genetic attenuation, biocontainment strategies, and programmable control systems is likely to expand the range of clinically acceptable indications [81,87], accelerating the incorporation of bacterial platforms into precision oncology as adjunctive and controllable therapeutic tools [85,88].

## 11. Clinical Translation and Future Perspectives

The transition from preclinical validation to clinical application remains the most critical stage in the development of bacteria- and toxin-based anticancer therapies. Although many approaches are still in early clinical phases, the translational landscape is steadily expanding [39]. Salinosporamide A, a marine bacterium-derived anticancer compound, has advanced to Phase III clinical trials for glioblastoma, representing one of the most clinically mature bacterial-derived agents in oncology [89]. In parallel, recombinant immunotoxins have demonstrated promising activity in Phase I and II trials for hematologic malignancies, including Hodgkin lymphoma [7,26].

Advances in synthetic biology are broadening the scope of bacterial systems beyond direct cytotoxicity. Genetically engineered bacteria have recently been developed as biological sensors capable of detecting tumor-derived DNA in vivo, highlighting their potential role in early cancer diagnosis and precision monitoring [90]. This dual diagnostic–therapeutic capability underscores the increasing versatility of bacterial platforms.

Collectively, the field has evolved from the empirical administration of live bacteria to the rational engineering of highly controllable and tumor-selective systems. The development of precision immunotoxins, stimulus-responsive oncolytic bacteria, and enzyme-activated prodrug strategies reflects significant maturation of bacterial bioengineering in oncology [20,90]. Moreover, emerging evidence indicates that modulation of the microbiome may enhance responsiveness to immunotherapy, suggesting that bacterial strategies can function not only as cytotoxic agents but also as immune modulators [23].

Despite these advances, widespread clinical translation remains limited by challenges including toxicity, immunogenicity, and biosafety concerns. Continued refinement of bacterial attenuation strategies, controlled gene-expression systems, and improved delivery specificity will be essential to overcome these barriers [91]. In addition, well-designed Phase II and III clinical trials are required to establish robust safety and efficacy profiles for these therapies [69].

In summary, bacteria and their toxins have progressed from experimental concepts to programmable therapeutic platforms with the potential to transform oncology through selective tumor targeting, immune activation, and controlled intratumoral delivery.

## 12. Conclusions

Bacteria and their toxins represent a promising class of biological agents for cancer therapy due to their ability to selectively colonize hypoxic and necrotic tumor regions that are poorly accessible to conventional treatments. Advances in synthetic biology and genetic engineering have enabled the development of engineered bacterial platforms, recombinant immunotoxins, and bacterial-directed enzyme prodrug therapies, expanding the range of strategies for targeted tumor destruction and immune activation. Despite encouraging preclinical and early clinical findings, important challenges remain, including safety concerns, immune clearance, pharmacokinetic limitations, and regulatory considerations. Continued progress in microbial engineering and translational research will be essential to optimize these approaches and facilitate the safe integration of bacteria-based therapies into future cancer treatment strategies.

## Figures and Tables

**Table 1:** Bacterial Toxins and Engineered Bacterial Platforms in Oncology: Mechanistic Diversity and Clinical Translation.

Bacterium/Toxin	Target Cancer Type	Mechanism of Action	Developmental Stage	Results	References
**I. Bacterial Immunotoxin**					
*Pseudomonas aeruginosa* exotoxin A (PE38)-based recombinant immunotoxin SS1P (SS1(dsFv)PE38)	Advanced malignant pleural mesothelioma (MSLN-positive).	After binding to mesothelin, SS1P undergoes receptor-mediated internalization. The *Pseudomonas* exotoxin A (PE38) catalytic domain inhibits protein synthesis via ADP-ribosylation of elongation factor-2 (EF-2), leading to tumor cell death.	Phase I clinical trial (frontline combination with pemetrexed + cisplatin).	Among 20 evaluable patients, 12 (60%) achieved partial response. At the maximum tolerated dose (45 µg/kg), 10 of 13 patients (77%) achieved partial response. Objective responses were associated with significant decreases in serum mesothelin, megakaryocyte potentiating factor (MPF), and CA-125 levels.	[36]
Diphtheria toxin-based fusion protein Tagraxofusp (SL-401)	Blastic plasmacytoid dendritic-cell neoplasm (BPDCN), an aggressive CD123 (IL3RA)–overexpressing hematologic malignancy.	IL-3 binds CD123 (IL3RA) → receptor-mediated internalization → diphtheria toxin catalytic fragment ADP-ribosylates elongation factor-2 (EF-2) → inhibition of protein synthesis → apoptosis.	Multicenter, open-label pivotal clinical study.	In previously untreated **BPDCN** patients receiving 12 µg/kg, combined complete response + clinical complete response rate 72% (95% CI 53–87); durable remissions observed. Capillary leak syndrome was the most clinically significant toxicity.	[37]
**II. Oncolytic bacteria**					
*Clostridium novyi*-NT (attenuated anaerobic spore-forming bacterium)	Treatment-refractory advanced solid tumors (intratumoral administration)	Intratumoral injection of *Clostridium novyi*-NT spores results in selective germination within hypoxic tumor regions, followed by bacterial proliferation and localized tumor tissue destruction.	Phase I clinical trial (3+3 dose-escalation design)	Spore germination and tumor colonization observed in a subset of patients; evidence of tumor necrosis; partial responses and disease stabilization reported; dose-limiting toxicities associated with inflammatory reactions.	[38]
*Salmonella enterica* serovar Typhimurium VNP20009 (purI−, msbB− deletions)	Metastatic melanoma (*n* = 23); metastatic renal cell carcinoma (*n* = 1)	Purl gene deletion (ΔpurI) confers adenine auxotrophy, restricting bacterial replication in normal tissues while permitting growth in purine-rich necrotic tumor microenvironments; msbB gene deletion (ΔmsbB) modifies lipid A structure, reducing TLR4-mediated inflammatory toxicity. After intravenous administration, the strain preferentially accumulates in hypoxic/necrotic tumor regions, inducing pro-inflammatory cytokine release (TNF-α, IL-1β, IL-6, IL-12) and tumor necrosis. Rapid systemic clearance limits sustained colonization.	Phase I (intravenous)	Intravenous administration of the attenuated strain was evaluated in a Phase I dose-escalation study for safety, tumor colonization, and systemic cytokine induction (IL-1β, TNF-α, IL-6, IL-12); no radiologically confirmed objective tumor responses were observed.	[39]
**III. Live Attenuated Immunotherapy**					
Live attenuated *Listeria monocytogenes* immunotherapeutic platform ADXS11-001 (Lm-LLO-E7)	Recurrent or advanced cervical carcinoma with confirmed Human Papillomavirus type 16 (HPV16) infection and active expression of the viral oncoprotein E7.	Preferential infection of antigen-presenting cells (APCs); truncated listeriolysin O (tLLO) mediates phagosomal membrane disruption and cytosolic escape; proteasomal processing of HPV16 E7 in tumors expressing the viral oncoprotein, followed by TAP-dependent loading onto MHC class I molecules; concurrent MHC class II presentation; activation of E7-specific CD8+ cytotoxic T lymphocytes and Th1-polarized immune response with increased interferon-gamma production; modulation of the tumor microenvironment, including reduction in regulatory T-cells (Tregs) and myeloid-derived suppressor cells (MDSCs).	Phase I and Phase II clinical trials	Phase I demonstrated safety and induction of HPV16 E7-specific cellular immune responses. Phase II reported clinical activity and survival benefit in patients with recurrent or advanced HPV16-positive cervical cancer.	[34,40,41]
**IV. Bacterial Toxin-based Cytotoxic Strategies**					
Cytolysin A (ClyA) Produced by *Escherichia coli* and *Salmonella enterica*. Delivered via engineered attenuated *Salmonella enterica* serovar Typhimurium strains.	Pancreatic cancer and other solid tumors (murine xenograft and orthotopic models)	ClyA is an α-helical pore-forming toxin that binds to cholesterol-rich membrane domains and undergoes conformational rearrangement leading to oligomerization and transmembrane pore assembly. This results in membrane permeabilization, ionic dysregulation (potassium efflux and calcium influx), mitochondrial dysfunction, caspase activation, and apoptotic or necrotic cell death depending on toxin concentration.	Preclinical only (in vitro and in vivo murine models). No human clinical trials reported to date.	Tumor-selective accumulation of engineered *Salmonella* expressing ClyA, significant inhibition of tumor growth in murine pancreatic models, stromal modulation, and increased immune cell infiltration within the tumor microenvironment.	[42,43]
*Clostridium difficile* toxin B (TcdB), large glucosyltransferase cytotoxin.	Pro-apoptotic glucosyltransferase cytotoxin with inflammatory modulation. Breast cancer (murine xenograft model).	TcdB enters target cells via receptor-mediated endocytosis and releases its glucosyltransferase domain into the cytosol, where it inactivates Rho family GTPases (Rho, Rac, Cdc42) through glucosylation. This disrupts actin cytoskeleton dynamics, impairs survival signaling pathways, induces mitochondrial dysfunction, activates caspase-dependent apoptosis, and modulates inflammatory mediators within the tumor microenvironment.	Preclinical only (in vivo murine models). No human clinical trials reported to date.	Recombinant TcdB significantly inhibited tumor growth in murine breast cancer models, reduced Bcl-2 expression, increased apoptosis, and altered inflammatory and signaling markers compared with controls.	[30]
*Bacillus anthracis* Lethal Toxin	MAPK pathway inhibition via LF metalloprotease activity. Melanoma; Neuroblastoma (murine xenograft models).	Binary toxin composed of Protective Antigen (PA) and Lethal Factor (LF). Following receptor-mediated internalization, LF—a Zn^2+^-dependent metalloprotease—cleaves MAPKs (MEK/MAPKK family), thereby inhibiting ERK, p38, and JNK signaling pathways that are critical for tumor cell proliferation and survival.	Preclinical (in vivo murine xenograft models). Antitumor efficacy demonstrated in melanoma and neuroblastoma models; no human clinical trials reported to date.	Systemic LT induced significant tumor growth delay and partial/complete regressions in human melanoma xenografts. Tumor growth inhibition was also observed in neuroblastoma xenografts and engineered LT variants in melanoma models.	[27,44,45]
*Clostridium perfringes* Enteroxin (CPE)	Claudin-3/4-mediated selective necrotic cytotoxicity Ovarian; Colorectal cancer	CPE is a pore-forming toxin that binds with high affinity to claudin-3 and claudin-4 tight junction proteins overexpressed in epithelial tumors. Binding promotes prepore and pore complex assembly, membrane destabilization, loss of ionic homeostasis, and selective necrotic/cytolytic death in claudin-overexpressing tumor cells.	Preclinical (in vivo murine xenograft and receptor-targeting models). No human clinical trials reported to date.	CPE and its C-terminal fragment (c-CPE) demonstrate preferential localization to claudin-3/4-overexpressing ovarian and colorectal tumor models, with selective cytotoxicity and tumor necrosis observed in preclinical studies.	[46,47,48]
**V. Bacterial Directed Enzyme Prodrug Therapy (BDEPT)**					
Genetically engineered *Bifidobacterium longum* expressing cytosine deaminase (CD)	Autochthonous rat mammary tumors; murine solid tumor models	Systemic administration results in selective colonization of tumor tissue. Expression of cytosine deaminase enables intratumoral conversion of 5-fluorocytosine (5-FC) into 5-fluorouracil (5-FU), leading to local cytotoxic effects within tumor tissue.	Preclinical validation (in vivo rodent models)	Demonstrated intratumoral production of 5-FU and significant tumor growth inhibition in rat mammary tumor models following administration of 5-FC.	[49,50]
Engineered anaerobic bacteria (suicide gene systems)	Solid tumors	Tumor-restricted enzymatic activation of prodrugs with bystander effect	Genetically engineered anaerobic or facultative anaerobic bacteria selectively colonize hypoxic/necrotic tumor regions and express prodrug-activating enzymes such as cytosine deaminase. Following systemic administration of a non-toxic prodrug (e.g., 5-fluorocytosine), the bacterial enzyme converts it locally into a cytotoxic agent (e.g., 5-fluorouracil), resulting in tumor-restricted drug activation and a bystander cytotoxic effect in neighboring tumor cells.	Preclinical (in vivo murine solid tumor and xenograft models). Demonstrated tumor localization, intratumoral prodrug conversion, and significant tumor growth inhibition. No definitive human clinical efficacy data reported for enzyme prodrug bacterial systems.	[51,52]

## Data Availability

The original contributions presented in this study are included in the article. Further inquiries can be directed to the corresponding author.
